# Arthroscopic‐assisted management of talus and calcaneus fractures: A narrative review of the clinical outcomes and surgical technique

**DOI:** 10.1002/jeo2.70510

**Published:** 2025-11-15

**Authors:** Hayden Hartman, Haley Tornberg, Paul Fine‐Lease, Arianna L. Gianakos

**Affiliations:** ^1^ Department of Orthopaedic Surgery University of Missouri Columbia MO USA; ^2^ Department of Orthopaedic Surgery Emory University Atlanta GA USA; ^3^ Middlebury College Middlebury VT USA; ^4^ Department of Orthopaedic Surgery, Yale Medicine Orthopaedics and Rehabilitation New Haven CT USA

**Keywords:** arthroscopy, calcaneus, fracture, minimally invasive, talus

## Abstract

While distinct in incidence and presentation, both talus and calcaneus fractures share a common challenge in their complex anatomy and high‐energy mechanisms resulting in difficulties in treatment. With traditional open reduction and internal fixation (ORIF), these fractures carry a high risk of complications, such as avascular necrosis, infection and post‐traumatic arthritis. New minimally invasive strategies have risen to the forefront, with arthroscopic‐assisted reduction and internal fixation (ARIF) emerging as a promising alternative. This technique offers the potential for enhanced visualisation, reduced soft‐tissue disruption, quicker recover and greater reduction advantage. The purpose of this narrative review was to discuss outcomes and surgical techniques of arthroscopic‐assisted reduction and internal fixation (ARIF) of talus and calcaneus fractures. ARIF techniques varied in patient positioning, portal use and fixation strategies, but consistently emphasised anatomic reduction via microscopic visualisation typically with temporary Kirshner wires, followed by fixation with cannulated screws. Despite the technical learning curve, the use of ARIF demonstrated clear advantages in minimising soft‐tissue trauma and enhancing joint preservation in both talus and calcaneus fractures. This review supports the growing role of minimally invasive techniques as a viable alternative to ORIF in patients with high‐energy talus and calcaneus fractures.

**Level of Evidence:**

Level IV.

**Abbreviations**
Abx: antibiotics
AOFAS: American Orthopaedic Foot and Ankle Society (Ankle‐Hindfoot Score)
APSF: arthroscopic assisted percutaneous screw fixation
ARIF: arthroscopic‐assisted reduction and internal fixation
ATFL: anterior talofibular ligament
AVN: avascular necrosis
CX: complications
DJD: degenerative joint disease
HR: hardware removal
K‐wire: Kirschner wire
LOE: level of evidence
MIS: minimally invasive surgery
ORIF: open reduction and internal fixation
PACO: percutaneous arthroscopic calcaneal osteosynthesis
PDSA‐CRIF: Percutaneous Distractor and Subtalar Arthroscopy Closed Reduction followed by Internal Fixation
PRISMA: Preferred Reporting Items for Systematic Reviews and Meta‐Analyses
PROM: patient‐reported outcome measures
SF‐36: 36‐Item Short Form Survey (Physical Component)
SP: screw pain
VAS: visual analogue scale
XR: X‐ray

## BACKGROUND

The calcaneus is the most fractured bone in the foot, accounting for over 60% of tarsal fractures [11, 39] and 2% of all adult fractures [32, 39]. While talus fractures are less common, representing less than 1% of all fractures and between 3% and 5% of fractures in the foot and ankle joint, both hindfoot injuries are complex injuries typically sustained in high‐injury traumas such as motor vehicle accidents or fall from height that pose a challenge to orthopaedic surgeons in terms of diagnosis and treatment [9, 14, 22, 39]. As a result of the traumatic aetiology of this injury, an estimated 70% of calcaneal fractures are intra‐articular, are often displaced with subtalar joint involvement and can be difficult to identify on plain radiographs [2, 11, 32, 39, 49, 63]. As a result, calcaneus fractures are often overlooked, resulting in a delayed presentation, worse outcome and higher risk of non‐union [45, 47]. These fractures can be devastating and are associated with a large sequela of complications, including heel widening, loss of heel height, malunion, early‐onset arthritis, stiffness, compartment syndrome and persistent pain with standing or walking [2, 9, 11, 14, 41, 49].

Like the calcaneus, the unique anatomy of the talus poses a significant challenge to orthopaedic surgeons in terms of treatment. Covered in articular cartilage, a network of ligamentous structures, minimal muscular attachments and lacking in abundant blood circulation, the talus is a difficult structure to visualise and reduce anatomically [8, 28, 41]. Additionally, the traumatic nature of these injuries often results in significant soft‐tissue damage and therefore must also be considered and addressed during treatment [40, 45, 47, 53, 57].

The approach to treatment has historically been quite controversial, with the best option still under debate [2, 8, 9, 11, 14, 28, 40, 41, 47, 49, 63]. Optimal treatment for these fractures is dependent on the fracture pattern, degree of displacement and the extent of articular involvement, and as such, treatment approaches should be taken on a case‐by‐case basis. However, there is a consensus that if surgery is indicated, early surgical intervention is preferred to achieve prompt restoration of bony anatomy [2, 11, 54, 56]. This approach is associated with a more favourable prognosis and is more cost‐effective than nonoperative treatment [8, 14, 59]. Both displaced calcaneus and talar body and neck fractures are traditionally treated by open reduction internal fixation (ORIF) [6, 24, 49, 54, 59, 63]. While ORIF enables direct visualisation of fracture fragments, it is associated with substantial risks – including soft tissue trauma, compromised blood supply, avascular necrosis (AVN), infection, and post‐traumatic arthritis, with complication rates reported as high as 65.4% for ankle arthritis, 34.6% for subtalar arthritis, and 38.5% for talar osteonecrosis [5, 7, 11, 39, 54, 63, 66]. Given these high complication risks, more minimally invasive options have gained popularity.

Arthroscopic‐assisted reduction and internal fixation (ARIF) has received increased attention as this technique visualises the fracture sites while simultaneously providing reduction and fixation with less violation of the blood supply and soft tissue, quicker recovery and lower rates of post‐traumatic arthritis [4, 5, 6, 7, 11, 21, 32, 34, 39, 52, 54, 63, 64]. Due to the rarity of talus and calcaneus fractures as well as the lack of consensus as to whether an arthroscopic approach should be considered over conventional ORIF, paucity of clinical data exists. The purpose of this narrative review was to discuss outcomes and surgical techniques of ARIF of talus and calcaneus fractures.

## INCLUDED STUDIES AND OUTCOMES

### Search strategy and data evaluation

In December 2023, a systematic review of the MEDLINE, EMBASE and Cochrane Library databases was performed between 2000 and 2024 based on the Preferred Reporting Items for Systematic Reviews and Meta‐Analyses (PRISMA) guidelines [38]. The following search terms were used for talus fractures: ‘(talus or talar) AND (arthroscopy or arthroscopic)’ and for calcaneus fractures: ‘arthroscopy AND calcaneus’, ‘arthroscopic AND calcaneal’, ‘arthroscopic AND calcaneus’ and lastly ‘arthroscopic AND calcaneal’. Inclusion and exclusion criteria are found in Table 1. Of note, there were no limitation parameters placed on study type for inclusion, aside from case reports being excluded. The titles, abstracts and full‐text articles were screened by two independent reviewers with a senior author resolving any disagreements. Data from each individual study were independently extracted and assessed by two independent reviewers, with a senior author resolving any conflicts. The primary outcome measures included clinical outcomes, complications and reoperations. Secondary outcomes included surgical technique. Meta‐analysis was not completed due to heterogeneity of studies. Subjective synthesis was completed due to lack of comparative cohorts and inability to obtain randomised studies from the available literature. There were no studies that completed direct comparison between ORIF and ARIF within the talus cohort, while two studies did so in the calcaneus cohort. Institutional review board approval was not sought for this study because protected health information was not required for this investigation.

**Table 1:** Inclusion and exclusion criteria.

Inclusion criteria	Exclusion criteria
Outcomes following arthroscopic treatment of talus fractures	Systematic reviews, conference abstracts, editorials, technique tips
Peer reviewed	Not peer reviewed
English language	Not English language
>1 patient per cohort	Unpublished studies
Published between 1/1/2000 and 12/31/2024	Animal, paediatric, cadaveric studies
Minimum 6‐month follow‐up	Less than 6‐month follow‐up

### Talus

**Figure 1.**
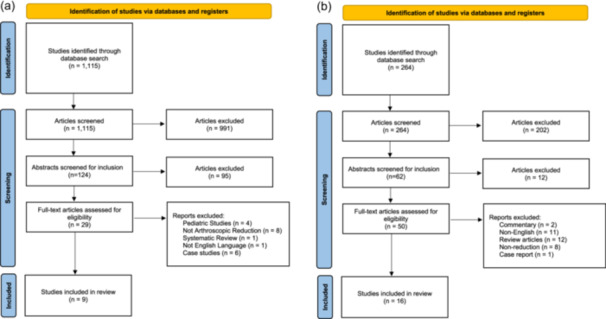
(a) Preferred Reporting Items for Systematic Reviews (PRISMA) flow diagram for talus fractures. (b) PRISMA flow diagram for calcaneus fractures.

The talus search generated 1115 studies, of which 9 met the inclusion criteria (Figure 1a and Table 2). A total of 157 patients (158 feet) underwent arthroscopic treatment for talus fracture. Included patients had a mean postoperative follow‐up time of 15.6 ± 12.4 months (range 6–36), were 33.3 ± 4.8 years (range 29–39), 86 patients (64.7%) were male, and at a mean body mass index of 24.3 ± 1.2 kg/m^2^ (range 23.5–25.1). Of the fractures classified via the Sneppen classification, 62 were Type II and 2 were Type III [22, 25, 66]. Of those utilising the Hawkins classification, 5 fractures were Type I, 27 were Type II and 3 were Type III [4, 55, 60]. The mean postoperative American Orthopaedic Foot and Ankle Score (AOFAS) was 88.1 ± 6.2 [4, 13, 22, 25, 51, 55, 65], indicating a good to excellent outcome. The mean postoperative visual analogue scale (VAS) was 0.82 ± 0.61 [60, 65, 66], demonstrating minimal pain. There was a total of four complications (2.53%; malunion‐1, subtalar arthritis‐3) and three secondary surgical procedures (1.90%; subtalar joint arthroscopy).

**Table 2:** Study characteristics, patient demographics, clinical outcomes and adverse events.

Author	LOE	Patients (*n*)	Feet (*n*)	Sex (m/f)	Age (y)	Follow‐up (mo)	Postop AOFAS	Postop VAS	Postop SF‐36	Complications	Secondary operations
**Talus**											
Jorgensen et al. [25]	4	2	2	2/0	29.5	12	93.5				
Bardas et al. [4]	4	4	4	1/3	38	18	92.75				
Wagener et al. [60]	4	7	7	3/4	39	26.4 ± 0.9		0.14			
Feng et al. [13]	4	48	48	30/18	32.21 ± 8.05	37.61 ± 9.85	96.85 ± 8.42		117.83 ± 9.25		
Yan et al. [66]	3	46	46	32/14	38.9 ± 7.9	6		1	90.59 ± 8.31	Malunion = 1	
Hu et al. [22]	4	15	16	10/5	31	36	85.7			Peri‐talar osteoarthritis = 2	
Xu et al. [65]	4	9	9	7/2	36.33 ± 9.77	21 ± 5.43	91 ± 3.16	1.33		Subtalar arthritis = 1	Subtalar Arthritis: 1
Sitte et al. [51]	5	2	2	1/1	29	6	80.5				
Sun et al. [55]	2	24	24	6/18	NA	NA	66.28 ± 7.51				Traumatic arthritis: 2
**Calcaneus**											
Gao et al. [16]	4	59	68	54/5	40.4	14	86.485	2.235		Transient sural nerve palsy = 1 Chronic foot pain = 2	None
Grun et al. [19]	4	25	26	23/2	44	15	85	2.05		Temporary nerve pain = 4	Screw removal = 2
Yoshimura et al. [69]	4	4	4	3/1	55.3	33.5	89			N/A	None
Hammond et al. [20]	4	14	17		‐‐	>3.0	NA			N/A	None
Pastides et al. [42]	4	30	33	20/10	39.6	24	72.18	2.95		Port site infection = 1, SP = 2	HR (prominent screw removal) = 2
Gavlik et al. [17]	4	15	15	16/1	41.1	14	93.7			Painful callosity over screw head =1	Screw removal and callosity excision = 1
Woon et al. [64]	4	22	22	17/5	43	33	84.2		77.7	SP = 1 Seroma = 1	Screw removal = 1
Law et al. [29]	4	14	15	13/1	51.4	79.2	90.3		92.7	SP = 2 Patient preference for removal = 1	HR = 3
Rammelt et al. [48]	4	24	24	21/3	42	29	92.1			Residual pain requiring intervention = 1 Conversion to open = 4 Prominent screw head = 2	HR = 2
Schuberth et al. [50]	4	24	24	22/2	45.3	56.1				Late DJD = 6	None
Sivakumar et al. [52]	4	9	9	7/2	44.9	17.9	87.8			SP = 1 Ongoing swelling = 1	Screw removal = 1
Yeap et al. [67]	3	14	15	10/4	42.1	16.9	86.7		57	Prominent screw = 1	Screw removal = 1
Rammelt et al. [46]	3	18	18	16/2	41.1	15	94.1			Painful callosity over screw head =1	Screw removal=1
Dai et al. [10]	3	52	52	34/18	61.7	23.7	94.6	1.4		‘Peritoneal tendon irritation= 3 Subtalar arthritis/SP = 2	HR = 2
Park et al. [41]	3	23	23	19/4	52.3	15.9	91.7	2.2	79.2	Transient sural nerve injury = 3 SP = 2	HR = 2
Yeomans et al. [68]	3	39	41	29/10	48.5	38.4				9 (2 wound cx, 1 SP)	HR = 1

Abbreviations: Abx, antibiotic; AOFAS, American Orthopedic Foot and Ankle Society (AOFAS) Ankle‐Hindfoot Score; CX, complications; DJD, degenerative joint disease; HR, hardware removal; m/f, male/female; mo, months; NA, not available; postop, postoperative; SF‐36, Physical Component of 36 item Short Form Survey; SP, screw pain; VAS, visual analogue scale; y, years.

### Calcaneus

The calcaneus search generated 264 studies, of which 16 met the inclusion criteria (Figure 1b and Table 2). A total of 423 patients (447 feet) successfully underwent arthroscopic assisted treatment for calcaneus fracture. Of the patients with reported sex, 81% identified as male, and 19% identified as female. The mean patient age was 46.2 ± 6.4 years (range 39.6–61.7). Of the fractures with reported Sanders classification, 12 (3%) were Sanders I, 282 (65%) were Sanders II, 130 (30%) were Sanders III and 7 (2%) were Sanders IV. Five studies reported Essex‐Loprestii classification, with 84 (54%) being joint depression and 72 (46%) being tongue‐type [16, 41, 52, 67, 68].

The AOFAS was the most utilised scoring tool, used in all but three studies [20, 50, 68], with a mean postoperative score of 88.3 (good to excellent outcome). The Bohler angle improved from a mean preoperative 8.1° to a mean postoperative 22.4°. In total, 47 complications (10.5%) were observed, of which the most common was symptomatic hardware in 14 fractures (3.1%). Nineteen patients (4.3%) required secondary procedures, all of which were to remove symptomatic hardware.

In a head‐to‐head study comparing ARIF and ORIF, ARIF was associated with significantly shorter postoperative hospital stay (*p* = 0.04) and quicker return to work (*p* = 0.00) compared to the ORIF group [67]. Both groups demonstrated significant improvement in Böhler's angle post‐intervention (ORIF: *p* = 0.003; ARIF: *p* = 0.002), with the ORIF group achieving a higher postoperative angle. Gissane's angle also significantly improved post‐intervention in the ARIF group (*p* < 0.001), while the change in the ORIF group trended toward significance (*p* = 0.05) [67]. No significant differences were observed between groups for duration of surgery, fracture union time, full weight bearing time, follow‐up duration, AOFAS scores, or SF‐36 physical and mental component scores.

A comparison of open, percutaneous, and arthroscopically assisted percutaneous (AA‐perc) fixation techniques found that the open approach resulted in significantly greater postoperative improvement in Böhler's angle compared to both percutaneous and AA‐perc methods (*p* < 0.05) [68]. However, the open group also had a descriptively higher rate of subsequent subtalar fusions, although no p‐value was reported. No significant difference in radiographic deformity correction was observed between the percutaneous and AA‐perc groups [68].

## SURGICAL TECHNIQUE

### Talus

Surgical technique was described by 14 studies (Table 3). Five studies positioned the patient prone using standard posteromedial and posterolateral portals. Eight studies positioned the patient supine. Seven of these utilised standard anteromedial and anterolateral portals. Wagener et al. accessed the ankle joint through a central‐medial portal for primary reduction and an additional sinus‐tarsi portal if preoperative X‐ray revealed fracture displacement into the subtalar joint [60]. One study used a semi‐prone swing position for portal access [66].

**Table 3:** Talus surgical technique.

Author	Total *N*	No. of feet	Positioning	Portals	Reduction
Jorgensen et al. [25]	2	2	Prone, tourniquet ipsilateral thigh	Posteromedial, posterolateral adjacent to calcaneal tendon	4 mm straight osteotome used to reduce the facture, Kirschner wire (K‐wire) stabilised fracture, calcaneal bone graft inserted, into subchondral fracture impaction void, 3.5 mm headless compression cannulated screw placed over K‐wire to achive compression and stabilisation Comminuted fracture: Dyotonics Powermini Small Joint Shaver chondrotome, osteotome, microprobe, straight punch, Pitbull Jr grasper debrided fx, 65 deg microfracture pick used on fx surface
Bardas et al. [4]	4	4	Supine, no external traction, tourniquet ipsilateral thigh	Anteromedial, anterolateral (debridement), 2 lateral subtalar portals anteroinferior to lateral malleolus (reduction)	Reduced by instrumental manipulation with help of hooked probe and trocar, with arthroscope in superior and inferior portals. Placed 2 K‐wires percutaneously to stablize fx for insertion of 2 4.5 mm cannulated double threaded compression screws for definitive fixation (talar neck)
Wagener et al. [60]	7	7	Supine, tourniquet ipsilateral thigh	Central‐medial portal sinus‐tarsi portal if fx was displaced and interposed in subtalar joint on preop XR	2.5 mm K‐wire placed distal to fx in medial aspect of talar head (second inserted in those where reduction was compromised by fragment interposition and a Hintermann distractor placed over the two K‐wires to remove interposed bone fragment), two 4.3 mm cannulated QWIX screws inserted to fix fx
Feng et al. [13]	48	48	Supine, affected leg on distal edge of operating table, tourniquet ipsilateral thigh, set at 60 kPA, additional axial traction and dorsiflexion may increase exposure	Standard anterolateral and anteromedial portals (debridement), anterior subtalar portal (ATFL and fx evaluate)	1.2 mm guide pin used to drill a tunnel 10 mm in at centre of footprint region through anterior subtalar portal, suture anchor inserted into tunnel, suture hook and PDS II suture passed through ATFL, tension adjusted, fx reduced, sutured anchor limbs in neutral ankle position
Yan et al. [66]	46	46	Semi‐prone, swing position, trunk to 30 deg, ankle joint extended back slightly, midfoot pushed back to complete reduction	Anteromedial and anterolateral approach for type IV and V fractures	K‐wires inserted from anterior to posterior, after an exchange rod inserted into posterior internal channel to peel off soft tissue, plane knife inserted to clear soft tissue behind joint capsule, 2 K wires placed parallel to second metatarsal bone, a K‐wire placed from anterior external channel to pry to fractured hfead and the ankle joint was extended back to complete the reduction of the body of talus, cannulated screws placed
Hu et al. [22]	15	16	Supine	Anteromedial (medial at junction b/w ankle joint and tibialis anterior tendon) and anterolateral portals (lateral to junction b/w anterior line of ankle joint and peroneus tertius tendon)	bone fragments, hematome removed using 4 mm cutter, straight microprobe, straight linvatec grasping forceps, fx fragments raised and reduced using microbe and osteotome. K‐wire embedded to temporarily stabilise (2.5 mm hollow screw used if there was a non‐constant bony fragment). flexor hallucis longus exposed, medially retracted, subtalar joint assesssed through posterior approach, fracture blocks temporarily fixed using K‐wire, XR used to ensure that guide pin located in middle of talar head, 4 mm hollow screws inserted, all K wires removed
Xu et al. [65]	9	9	Prone, tourniquet ipsilateral thigh	Posteromedial, posterolateral	Through the medial portal, the fracture is temporarily reduced by compression with a probe and then fixe with a 1.5 mm K‐wire, followed by a 2.5 mm K‐wire into the subtalar joint to reduce the posterior talus. When satisfactory reduction is achieved, coordination with computer software is achieved to allow for robotic arm to place 4.0 mm screws for fixation.
Sitte et al. [51]	2	2	Prone, tourniquet ipsilateral thigh	Posteromedial and posterolateral, central and anterolateral (for subtalar joint)	Pressurised irrigation system used. Subtalar arthroscopy completed. Two wires placed parallel to each other through posterolateral portal; entry for medial wire was lateral tubercle of talus and lateral wire was insertion of posterior talofibular ligament. Third wire inserted to stablize the fracture temporarily. Two 3.5 mm screws inserted. Wires removed. Drain placed in posterolateral portal for 24 hours.
Sun et al. [55]	24	24	Supine, tourniquet ipsilateral thigh	Anteromedial approach	Reduction visualised through arthroscope and stabilizied with two to three 1.5–2.0 mm diameter K‐wires. Two‐four countersunk screws confirmed fixation under C‐arm

Fracture reduction utilised various instrumentation: an osteotome [22, 25], a probe [4, 12, 22, 36, 37, 65], Kirschner wire(s) (K‐wires) [15, 36, 51, 60], manual manipulation [35, 66] and a freer elevator [26]. Cannulated screws were the primary fixation method in 14 studies, with one study using K‐wires for both reduction and fixation, which were removed seven weeks post‐operatively. Postoperative casting for 6–8 weeks with partial weight‐bearing was completed in all studies.

### Calcaneus

Fifteen of the studies evaluated in this review reported on patient positioning (Table 4). Thirteen studies reported that patients were preferentially placed in the lateral decubitus position during the procedure [10, 17, 20, 29, 41, 42, 46, 48, 52, 64, 67, 68, 69]. Hammond et al. specified that patients were preferentially placed in the supine position if there were bilateral injuries or concern for spine injury [20]. Pastides et al. specified that patients were placed prone when bilateral injuries were present [42]. Two studies preferentially positioned patients in the prone position [16, 19], and one study preferentially placed patients supine [50]. Two to three portals were utilised for articular assessment, reduction, and fixation. The anterolateral, posterolateral and middle portals were the most used, with the posterolateral and sinus tarsi portals typically added for increased visualisation. Most of the studies used a mixed approach combining several different portals, including anterolateral and posterolateral portals.

**Table 4:** Calcaneus surgical technique.

Study	Technique	Portal placement	Portals used	Patient position
Gao et al. [16]	PDSA‐CRIF	Posterolateral and posteromedial portals for posterior ankle and subtalar arthroscopy. ± sinus tarsi portal if additional visualisation was needed	2.7 mm	Prone
Grun et al. [19]	PACO	Anterolateral portal	4.0‐mm, 30‐degree,knee arthroscope	Prone
Yoshimura et al. [69]	LACO	Anterolateral, middle and posterolateral	2.7‐mm, 30‐degree scope	Lateral decubitus
Hammond et al. [20]	Percutaneous	Posterior para‐achilles or lateral	not specified	Lateral (or supine if concerns for spine injury or fractures were bilateral)
		sinus tarsi portals		
Pastides et al. [42]	PACO	Two sinus tarsi portals	4.0‐mm, 30‐degree,knee arthroscope	Lateral for unilateral, prone for bilateral
		and often a third posterolateral portal		
Gavlik et al. [17]	PACO	Posterolateral portal but changed to anterolateral portal for better visualisation in some cases	1.9 mm/0 degrees or 4.0 mm/30 degree	Lateral decubitus
Woon et al. [64]	Dual‐modality (fluoroscopy + arthroscopy) for all fractures	Anterolateral and midlateral portals	2.4 mm, 0 degree scope	Lateral decubitus
Law et al. [29]	PACO	Anterolateral and midlateral portals,	2.4 mm, 0‐degree wrist arthroscope	Lateral decubitus
Rammelt et al. [48]	PACO	Anterolateral or posterolateral portal ± an additional middle portal from lateral	2.7 mm, 30 degree	Lateral decubitus
Schuberth et al. [50]	Arthroscopy for medial fractures, incision for lateral fractures	Lateral sinus tarsi portal	4.0‐mm 70 degree arthroscope	Supine
Sivakumar et al. [52]	One‐point distraction and arthroscopy	Anterolateral portal, middle portal	2.9‐mm 30‐degree arthroscope	Lateral decubitus
Yeap et al. [67]	APSF	Anterolateral, central and posterolateral portals	2.7 mm 30‐degree forward facing arthroscope	Lateral decubitus
Rammelt et al. [46]	PACO	Anterolateral or posterolateral portal	1.9 mm, 0 degrees	Lateral decubitus
Dai et al. [10]	Subtalar arthroscopy			Lateral decubitus
	with medial calcaneal‐talar joint			
	distraction			
Park et al. [41]	Evaluation of fracture reduction using fluoroscopy + arthroscopy (group 2) to assist in open approach	Reduction evaluated via open subtalar dry arthroscopy through the previous skin incision or additional anterolateral portal. Sinus Tarsai approach	(2.4‐mm) arthroscope	Lateral decubitus
Yeomans et al. [68]	Arthroscopically assisted percutaneous approach	Two sinus tarsi portals	30‐degree, 4.0‐mm knee arthroscope	Not listed

Abbreviations: APSF, Arthroscopic Assisted Percutaneous Screw Fixation; PACO, Percutaneous Arthroscopic Calcaneal Osteosynthesis; PDSACRIF, Percutaneous Distractor and Subtalar Arthroscopy Closed Reduction followed by Internal Fixation.

Intraarticular reduction was successfully completed using a manipulation technique, either with a percutaneous joystick, self‐made reduction devices with a Steinmann pin inserted into the calcaneal tuberosity, K‐wires, or a periosteal elevator to reduce the displaced facets. Temporary fixation was achieved using K‐wires, with permanent fixation achieved using 1–5 cannulated screws, typically inserted lateral to medial, parallel to the posterior facet. Each study ensured screw placement into the sustentacular fragment if applicable, and the choice to use washers with the screws varied based on surgeon preference. After adequate anatomic reduction was achieved and confirmed with direct visualisation using the arthroscope, fluoroscopy, or both, the fractured fragments can be fixated using the appropriate screws. Postoperatively, patients were immobilised for the first 6–8 weeks, transitioned to partial weight bearing from 8 to 10 weeks, and allowed to fully weight‐bear at 10–12 weeks.

## DISCUSSION

Recent literature has demonstrated an expansion of the use of minimally invasive techniques for the treatment of numerous pathologies, including calcaneal fracture reduction and fixation. This shift stems from the inherent dissatisfaction with the high complication rate seen following ORIF for calcaneal and talus fractures. While open procedures like ORIF have historically been the more commonly utilised approach in the management of these fractures, minimally invasive approaches like ARIF have come into favour recently, as they have been reported as having a lower complication profile and quicker recovery time comparatively [6, 11, 20, 21, 32, 34, 39, 52, 54, 64]. This lower complication rate is likely due to the smaller incisions and subsequent lower infection risk associated with arthroscopic intervention when compared to the relatively large incision required for ORIF. Though numerous studies report similar clinical and radiographic outcomes between ARIF and ORIF, direct comparison is limited by the fact that ORIF is often reserved for more severe injuries with greater soft tissue compromise, which inherently predisposes to worse outcomes. Anecdotally, minimally invasive interventions can result in less soft tissue stripping and less pain and swelling for the patient. This allows for a quicker return to activities of daily living, a quicker return to work or activity, and fewer opioids required during the postoperative period [3]. Beyond the recovery potential, arthroscopic techniques offer potential advantage in achieving greater visualisation to achieve higher reduction accuracy – a crucial element to minimising post‐traumatic arthritis. Although early outcomes following ARIF are promising, long‐term data on functional outcomes, arthritis progression, and implant survivorship remain limited and warrant further investigation.

This study also evaluated the various surgical techniques described when performing ARIF for calcaneal and talus fractures. Although the use of arthroscopy for reduction of fractures has been shown to require a learning curve, this technique affords adequate exposure without the use of a large incision, decreased local trauma, and improved assessment of concomitant lesions, while minimising postoperative complications and improving fracture healing and an expedited return to baseline [23, 44]. Arthroscopic intervention enables irrigation and debridement of the joint, effectively clearing debris and haematoma to enhance articular visualisation. Clear assessment of the articular reduction relies heavily on the ability to view the subtalar joint. However, achieving a proper view can be challenging due to the joint's narrow and complex anatomy. Different approaches have been demonstrated with success in technical articles published in the literature.

### Clinical relevance

#### Talus

Talus fractures, often high‐impact traumatic injuries, pose surgical challenges due to the poor vascular supply and complex ligamentous network compromising visualisation [41]. ORIF, the conventional standard treatment, has reported a 34%–65% complication rate including AVN, vascular and soft tissue damage [5, 7]. Recently, ARIF has gained favour for being a less invasive, lower complication, higher visualisation approach [15, 18, 30]. ARIF grants adequate exposure of the talus for correction, while simultaneously minimising complications through smaller incisions, providing both diagnostic and treatment value by identifying associated lesions and loose bodies, evacuating hemarthrosis, and joint debridement [15, 18, 30, 60].

When assessing ARIF, various patient‐reported outcome measures (PROM) and scoring systems were utilised to determine post‐operative pain and satisfaction. This study demonstrated that postoperatively, all PROMs demonstrated excellent outcomes for both talus and calcaneus fractures utilising ARIF. Postoperative complication evaluation is crucial to determine the efficacy of ARIF. With ORIF, Type II talar neck fractures have a reported AVN rate of 20%–50%, with an approximate 10% incidence of nonunion or delayed union [18, 30, 35, 58, 61, 62]. The current study's findings reveal a complication rate of 2.53%, with complete avoidance of AVN and minimised lack of union (0.63%). Similarly, post‐traumatic arthritis is a primary sequela after ORIF for talar fractures (13%) [1, 31]. Within a similar follow‐up period, this study demonstrated a 1.90% rate of arthritis through a minimally invasive approach. These findings lend more credence to previous literature stating that ORIF is locally traumatic and aggressive, and the use of a minimally invasive technique can reduce these risks. Additionally, most patients (1.90% reoperation rate) did not require any further treatment aside from their initial fracture fixation. No secondary complications were introduced by the arthroscope, further supporting its benefit. The use of arthroscopy as both a primary technique or assistive adjuvant for articular reduction visualisation is advantageous for both exposure and complication minimisation.

#### Calcaneus

This study demonstrated good clinical outcomes, improved radiographic measures and low complication rates following ARIF for calcaneus fractures. While this literature review does not include a comparative group, several studies have demonstrated that, amongst their own comparator groups, there is no difference in radiographic outcomes, including Bohler angles and degree of step‐off at final follow‐up in patients undergoing ARIF when compared to patients who underwent ORIF [29, 32, 41, 67]. This study also found a very low aggregate complication rate of 10.5% using ARIF to repair calcaneal fractures. The most reported complication was symptomatic hardware, with the most common reason for reoperation being removal of the symptomatic hardware. It is important to note that 2 studies included in this review were comparative in design. When comparing ARIF to ORIF, Yeap et al. found that, while patients had comparable clinical and radiographic outcomes, those who underwent ORIF had a significantly longer hospital stay by 3.5 days and took significantly longer to return to work compared to the ARIF patients, requiring an extra 3.3 months of recovery [67]. In a comparison between open versus percutaneous versus arthroscopic‐assisted percutaneous reduction methods, Yeomans et al. demonstrated that, while the open approach allowed for a greater improvement in postoperative Bohler angles compared to both percutaneous and arthroscopic‐assisted percutaneous approaches, it was also associated with a higher percentage of subtalar fusions [68]. Moreover, they noted that there was no difference in deformity correction when comparing arthroscopic‐assisted percutaneous fixation to percutaneous fixation alone. Studies have demonstrated that patients who underwent ARIF have no difference in clinical outcomes, including range of motion, and patient‐reported outcomes on VAS, AOFAS and SF‐36 at final follow‐up when compared to patients who underwent ORIF [29, 32, 41, 67].

### Pearls and pitfalls

#### Talus

When considering a surgical technique, it is crucial to weigh advantages and disadvantages (Table 5). Talus ARIF is a technically demanding procedure requiring ankle arthroscopy knowledge, accompanied by a learning curve [22, 60]. However, positioning is standard – either supine with anterior portals or prone with posterior portals – and can be reproduced in a homogenous fashion for stable fixation [61]. Use of sinus tarsi and lateral portals is to be considered for further subtalar assessment, often following reduction. Should the fracture present differently than preoperative radiography, fixation alternatives are limited and conversion to open procedures can be challenging due to impaired exposure from fluid extravasation [25]. Identification of concomitant lesions, loose bodies, assessment of cartilage injuries, as well as adequately visualised fracture treatment without additional osteotomy for exposure are all pearls of ARIF [22]. ARIF is minimally invasive, reducing AVN risk because the damage to local blood supply is minimised or entirely spared when compared to ORIF [60]. The accessory portals allow for subtalar joint evaluation, in turn avoiding a step‐off, removing intra‐articular debris, which overall minimises the future articular degeneration and traumatic arthritis development [60]. ARIF is best suited for displaced talus fractures that are difficult to visualise with fluoroscopy alone, as arthroscopy provides superior visualisation of the joint space [15]. Additional portal sites and/or distraction of the joint, often via dorsiflexion, is recommended if visualisation difficulty persists [15, 33]. Martin et al. emphasise positioning of the arthroscope lateral to the flexor hallucis longus for neurovascular protection [33]. Probe or additional instrumentation can be beneficial to protect and isolate critical structures, such as the tibial nerve and posterior tibial artery [65]. Cannulated screw placement accuracy is also enhanced with the use of arthroscopy prior to C‐arm fluoroscopy confirmation [51].

**Table 5:** Pearls and pitfalls.

Pearls
Avoid need for medial or lateral osteotomies for exposure/access
Posteromedial and posterolateral portals are adjacent to the calcaneal tendon
Standard anteromedial and anterolateral for anterior approach
Lateral subtalar portals anteroinferior to lateral malleolus
Patient prone (posterior), supine (anterior)
Additional axial traction and dorsiflexion may increase exposure
Lateral calcaneus bone graft (percutaneously harvested) can assist fx fixation
Lower risk of avascular necrosis
Minimally invasive surgery – less local soft‐tissue damage, preserves local blood supply
Less damage to articular cartilage
Probe can be utilised to avoid tibial nerve and the posterior tibial artery
Superior visualisation of joint space when combined with fluoroscopy compared to fluoroscopy alone
**Pitfalls**
New procedure, potentially longer operative/tourniquet times
Conversion to an open procedure may be difficult to achieve
ARIF may not be an option once arthroscopically evaluated
Difficulty navigating narrow joint space with arthroscope requires learning curve, if not done correctly may result in higher complications
Risk of using the posterior portal results in higher risk of contact and potential subsequent damage to the sural nerve and surrounding subchondral bone

#### Calcaneus

The subtalar joint space is extremely narrow, and the calcaneus is irregularly shaped with several different facets, which may pose increased difficulty in achieving adequate reduction using traditional‐sized arthroscopes and thus a steeper learning curve in mastering this technique [44]. If not done correctly, this may be associated with a higher risk of rare but devastating complications, including damage to the neurovascular and ligamentous structure,s as well as compartment syndrome in the setting of excessive fluid extravasation [44].

Proper patient positioning and portal selection are critical for successful subtalar arthroscopy. The lateral decubitus position is most commonly achieved [10, 17, 20, 29, 41, 42, 46, 48, 52, 64, 67, 68, 69] though prone positioning with 90° knee flexion and calcaneal skeletal traction improves visualisation by 45% [27, 43]. Joint access is typically achieved through lateral or posterior approaches. The sinus tarsi approach is favoured for its minimally invasive nature and low neurovascular risk but provides limited visualisation, increasing the likelihood of non‐anatomic reduction. The lateral approach remains the preferred method for assessing subtalar pathologies and is commonly performed using a two‐ or three‐portal technique, with anterolateral, posterolateral, and middle portals most frequently utilised [10, 27, 43]. Posterolateral portals enhance posterior facet visualisation and allow assessment of screw penetration, but they carry a higher risk of sural nerve injury and subchondral bone damage [46]. Arthroscope selection plays a crucial role in optimising visualisation. While most studies favour 2.4–2.9 mm scopes, the 1.9 mm semi‐rigid arthroscope provides superior manoeuvrability in the narrow joint space, aiding precise anatomic reduction [44]. Schanz pins can be used to apply varus stress on the subtalar joint, further facilitating exposure [46]. However, careful patient selection is necessary, as injury severity influences the feasibility of this technique.

While studies have shown promising results, they have primarily been focused on Sanders II and III fractures. Arthroscopic assistance may not be the ideal choice in highly comminuted fracture patterns where the fracture fragments are deeply impacted, given the associated difficulty of reduction and the associated sequela of non‐anatomic reduction. However, if this technique is implemented with proper training and equipment, the benefits of arthroscopic‐assisted reduction may yield superior patient‐reported outcomes, radiographic outcomes, and clinical results when compared to traditional techniques in individual, comparative studies [10, 16, 17, 19, 20, 29, 41, 42, 46, 48, 50, 52, 64, 67, 68, 69].

### Limitations

This study acknowledges inherent limitations, such as study heterogeneity limiting cross‐sectional analysis. Data collection and extraction was done by two independent reviewers, rather than blindly. Furthermore, there were varied mechanisms of injury, surgical approaches, follow‐up times, and procedure novelty across studies. The small sample size, short duration of follow‐up, and lack of long‐term outcome evaluation inevitably introduced bias. The aforementioned aspects present preconception, though analysis was pursued as objectively as possible. Further research is required directly comparing open, arthroscopic and percutaneous talus fixation to determine outcomes and indications amongst varying fracture patterns and patient populations.

## CONCLUSION

This study demonstrates ARIF for talus and calcaneus fractures results in good clinical outcomes with excellent AOFAS scores and minimal postoperative VAS pain scores, as well as low complication rates (2.5% for talus; 10.5% for calcaneus). These findings indicate that the use of arthroscopy introduces a beneficial diagnostic tool for joint assessment as well as an adjuvant to achieve adequate reduction and visualisation. The results of this investigation should be utilised to develop randomised control or prospective cohort studies evaluating long‐term outcomes and indications of ARIF compared to ORIF for both talus and calcaneus fracture fixation.

## AUTHOR CONTRIBUTIONS

All authors contributed to the study conception and design. **Hayden Hartman**: Conceptualisation, methodology, formal analysis and investigation, writing – original draft preparation, writing – review and editing. **Haley Tornberg**: Conceptualisation, methodology, writing – original draft preparation, writing – review and editing. **Paul Fine‐Lease**: Methodology, formal analysis and investigation, writing – review and editing. **Arianna L. Gianakos**: Conceptualisation, methodology, writing – original draft preparation, writing – review and editing.

## CONFLICT OF INTEREST STATEMENT

The authors declare no conflicts of interest.

## ETHICS STATEMENT

This research did not receive any specific grant from funding agencies in the public, commercial, or not‐for‐profit sectors. Ethical approval was not sought for this study as we did not access any patient records.

## Data Availability

Data sharing not applicable to this article as no datasets generated or analysed during the current study.
